# RNA modifications and epigenetic regulation in plants

**DOI:** 10.1016/j.abiote.2026.100020

**Published:** 2026-01-12

**Authors:** Xinran Zhang, Jiangbo Wei

**Affiliations:** a Department of Chemistry, National University of Singapore, Singapore, 117544, Singapore; b Department of Biological Sciences, Cancer Science Institute, National University of Singapore, Singapore, 117544, Singapore

**Keywords:** Chromatin-associated RNAs, *N*^6^-methyladenosine, Plant chromatin regulation, Epitranscriptome

## Abstract

Chemical modifications of RNA, such as *N*^6^-methyladenosine (m^6^A), have emerged as critical regulators of gene expression and chromatin dynamics in eukaryotes. Research on RNA modifications in plants has primarily focused on mRNAs and their post-transcriptional roles. Recent studies in mammals have shown that various chromatin-associated RNAs (caRNAs) regulate chromatin structure and transcription, but the presence and functions of plant caRNAs remain largely unexplored. This review systematically introduces the current mechanisms and detection methods of RNA modifications and their roles in epigenetic regulation, with a specific focus on caRNAs in plants. Finally, we offer future perspectives, emphasizing that a deeper understanding of the epitranscriptomic regulation of caRNAs will be essential for decoding plant chromatin dynamics and may open new avenues for crop improvement.

## 1 Introduction

Chemical modifications of RNA, particularly m^6^A, have emerged as essential post-transcriptional regulators of RNA metabolism and gene expression in eukaryotes [[1], [2], [3], [4]]. These modifications are dynamically installed, removed, and interpreted by dedicated proteins known as writers, erasers, and readers, respectively [[5], [6], [7]]. In mammals, m^6^A deposition is mediated by the METTL3-METTL14 methyltransferase complex [[8], [9], [10]], removed by demethylases such as FTO [[11], [12], [13], [14]] and ALKBH5 [15], and recognized by YTH domain-containing proteins that influence RNA splicing, translation, and decay [16,17]. Homologous pathways exist in plants: MTA, MTB, and FIP37 function as the core of the methyltransferase complex [[18], [19], [20], [21], [22]], while ALKBH9B and ALKBH10B serve as demethylases with roles in flowering and stress responses [18,19,21,[23], [24], [25]]. YTH domain-containing proteins, such as ECT1-8 and CPSF30-L [[26], [27], [28], [29]], act as m^6^A readers and contribute to development, immunity, and plant hormone signaling.

Epigenetic regulation in plants encompasses a complex interplay of DNA methylation, histone modifications, and chromatin remodeling, collectively shaping transcriptional landscapes and genome stability. Unlike animals, plant DNA methylation occurs not only in the CG but also the CHG and CHH contexts [[30], [31], [32]], which is maintained by MET1, CMT2/3, and DRM2, respectively [33,34] and established *de novo* via the RNA-directed DNA methylation (RdDM) pathway [30,31,33]. Histone modifiers such as SET-domain proteins [[35], [36], [37]] and Jumonji (JMJ) demethylases [[38], [39], [40], [41]] regulate developmental timing, flowering, and stress responses through histone H3K4 and H3K27 methylation dynamics. In addition, histone acetylation is dynamically regulated by histone acetyltransferases (HATs) [42,43] and histone deacetylases (HDACs) [[44], [45], [46]]. Chromatin remodelers such as the DDM1 and SWI/SNF complexes facilitate nucleosome repositioning and epigenetic silencing, particularly at transposable elements and heterochromatic regions [[47], [48], [49]]. These interconnected pathways ensure proper developmental transitions and environmental plasticity in plants.

Chromatin-associated RNAs (caRNAs), including long non-coding RNAs (lncRNAs), R-loops, and small RNAs, play essential roles in epigenetic regulation in plants. lncRNAs function in developmental processes such as vernalization and environmental responses by recruiting Polycomb complexes or the DNA methylation machinery to specific loci [[50], [51], [52], [53]]. R-loops, which form co-transcriptionally, can help establish distinct chromatin environments and structural features, thereby dynamically modulating gene expression [49]. Small interfering RNAs (siRNAs), which are produced through the RdDM pathway, guide *de novo* DNA methylation and reinforce transcriptional silencing across transposable elements and repetitive sequences [33]. While the regulatory functions of these caRNAs have been increasingly recognized, whether they carry RNA modifications, and how such modifications affect their roles in regulating plant chromatin, remain largely unexplored.

Recent studies in animal systems have demonstrated that chemical modifications on caRNAs can actively shape chromatin states and transcriptional activity [54]. m^6^A installed by METTL3 on caRNAs can be recognized by YTHDC1, which recruits the nuclear exosome to degrade these transcripts and reduce chromatin accessibility [55,56]. Conversely, FTO-mediated m^6^A demethylation stabilizes caRNAs and prevents heterochromatin formation at specific loci [13]. In addition to regulating RNA decay, m^6^A reader proteins can also recruit the DNA demethylase TET1, thereby linking RNA methylation on caRNAs to active DNA demethylation and chromatin remodeling [57,58]. Additionally, 5-methylcytosine (m^5^C) on caRNAs can be recognized by MBD6, which interacts with polycomb repressive deubiquitinase complexes (PR-DUBs) to maintain transcription [59]. These findings highlight the roles of RNA modifications as a critical regulatory layer in the interplay between caRNAs and the epigenome.

## 2 RNA modifications in plants

To date, most studies of RNA modification in plants have focused on m^6^A, primarily on mRNA, with several major research directions established. These include investigating the dynamics of mRNA m^6^A levels in plants during different developmental stages and under various environmental stress conditions and examining their impacts on gene expression and plant physiology; elucidating the functions of mRNA m^6^A-associated enzymes, including methyltransferases, demethylases, readers, and associated cofactors; exploring the interplay between mRNA m^6^A modification and other epigenetic mechanisms, such as DNA methylation and histone modifications; and developing advanced technologies for the high-resolution detection of mRNA m^6^A, including applications at the reproductive cell, specialized cell, and single-cell levels.

### 2.1 Dynamics of m^6^A levels in plant mRNAs

In mRNA, m^6^A is predominantly enriched near the 3′ untranslated region (3′ UTR) and around stop codons, a distribution pattern generally conserved in plants [[60], [61], [62], [63], [64]]. In *Arabidopsis* (*Arabidopsis thaliana*) and rice (*Oryza sativa*), most m^6^A sites are highly enriched within 3′ UTRs, followed by coding sequences and 5′-untranslated regions (5′ UTRs) [61,65,66]. Notably, in *Arabidopsis*, the distribution of m^6^A varies across developmental stages; for example, floral tissues exhibit increased m^6^A levels along the coding sequence and reduced enrichment near the 3′ UTR compared to leaves [67].

Early efforts to measure m^6^A abundance in plants relied on low-throughput quantitative techniques. Thin-layer chromatography (TLC) was used to quantify m^6^A levels in *Arabidopsis*, revealing tissue-specific differences in m^6^A/A ratios: approximately 0.35 % in seeds, 0.5 % in roots, 0.9 % in leaves, and 1.3 % in inflorescences. These results indicate that reproductive tissues exhibit higher levels of m^6^A modification compared to vegetative organs [19]. These measurements represent early bulk-level assessments, laying important groundwork for understanding tissue-specific patterns of m^6^A deposition.

A subsequent transcriptome-wide m^6^A profiling study revealed that a substantial proportion of expressed genes in *Arabidopsis* are modified by m^6^A. Specifically, 70.6 % of transcripts in leaves, 73.7 % in flowers, and 76.7 % in roots carried m^6^A marks; these results are significantly higher than previous estimates in animals or plants. The m^6^A/A ratio within modified transcripts ranged from 0.44 % to 0.61 %, while the average ratio across the whole transcriptome was estimated to be 0.35 %–0.50 % in all three organs [68].

A comprehensive *Arabidopsis* m^6^A atlas was recently generated using MeRIP-seq across 100 samples, representing diverse tissues, developmental stages, and environmental stimuli. The study profiled 12,000–15,000 m^6^A peaks per sample and revealed consistent enrichment near stop codons, confirming earlier findings. The majority of m^6^A sites contained the conserved RRACH motif. This dataset covers over 90 % of previously identified m^6^A-bearing genes and includes 4279 new targets [69]. Comparative profiling of differentiated callus and leaf tissues in rice identified 8138 and 14,253 m^6^A-modified genes, respectively. Notably, 1792 and 6508 genes were tissue-specific m^6^A-modified genes in callus and leaf tissue, respectively, with a substantial portion (∼5500 in leaf) selectively methylated despite active expression in both tissues [70].

**Fig. 1:**
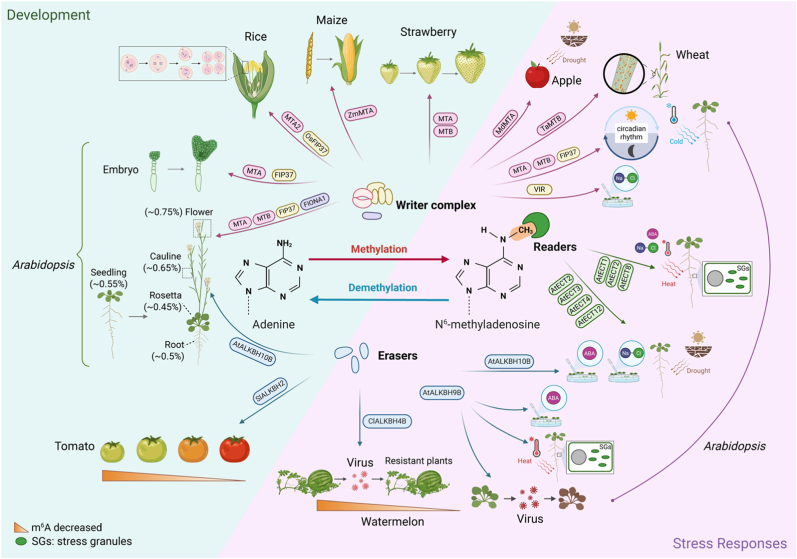
Conserved and diversified functions of m^6^A RNA methylation in plant development and stress responses. The diagram summarizes the results of studies on m^6^A in several plant species. Core writer components (MTA, MTB, FIP37, VIR) and erasers (e.g., ALKBH family proteins) mediate the dynamic regulation of m^6^A during key developmental processes such as embryogenesis, flowering, fruit ripening, and circadian rhythms. m^6^A levels vary across tissues and stages, with generally higher abundance in reproductive tissues. Reader proteins (e.g., ECTs) interpret m^6^A marks to influence transcript stability, translation, and localization. Under stress conditions, m^6^A modifications participate in ABA signaling, salt and cold responses, stress granule formation, and antiviral defense. Figure was created with BioRender.com.

Recent m^6^A-SAC-seq analysis provided high-resolution m^6^A maps across developmental stages in both rice and *Arabidopsis*. In total, 205,691 m^6^A sites were identified across 22,574 genes in rice, and 188,282 sites were identified across 19,984 genes in *Arabidopsis*. Many of these sites are conserved between orthologous genes in the two species, particularly those involved in tissue development, photosynthesis, and stress responses, while species-specific differences also suggest divergence in m^6^A regulatory networks [66] (Fig. 1). In tomato (*Solanum lycopersicum*), a marked decrease in global m^6^A levels was observed during fruit ripening, further supporting the notion that m^6^A modification is tightly regulated during plant development [62] (Fig. 1).

Together, these findings demonstrate that m^6^A is a conserved, reversible, dynamically regulated RNA modification that plays vital roles in regulating gene expression, plant development, and adaptation to environmental changes.

### 2.2 m^6^A-associated effector proteins

m^6^A is dynamically regulated in plants across developmental stages, tissue types, and in response to various types of environmental stress, with diverse functional impacts. This dynamic nature is orchestrated by effector proteins comprising methyltransferases (writers), demethylases (erasers), and m^6^A-recognizing proteins (readers) [71]. The writer of m^6^A is the methyltransferase complex (MTC), consisting of core methyltransferase-like 3 (METTL3) and methyltransferase-like 14 (METTL14). In mammals, this complex comprises METTL3, METTL14, WTAP, VIRMA, HAKAI, RBM15, RBM15B, and ZC3H13 [8,9,72]. METTL3 and METTL14 possess MT-A70-like domains and form a heterodimer, where METTL3 serves as the catalytic core, utilizing S-adenosylmethionine (SAM) as the methyl donor, and METTL14 provides RNA-binding specificity [73].

The m^6^A methyltransferase complex is evolutionarily conserved between animals and plants. In *Arabidopsis*, MTA and FIP37, homologs of mammalian METTL3 and WTAP, respectively, are essential for embryogenesis. T-DNA mutants of either gene show embryo-lethal phenotypes with developmental arrest at the globular stage, which is consistent with findings in animals and yeast [18,19,21]. MTA, MTB, and FIP37 also interact with the blue-light receptor CRY2 to form phase-separated condensates, thereby regulating the circadian rhythm in an m^6^A-dependent manner [74]. In rice, OsFIP and OsMTA2 play critical roles in reproductive development. *osfip* mutants show defective anther formation and reduced fertility due to abnormal meiosis [63]. OsMTA2 further influences growth and pollen development by interacting with translation initiation factors to regulate target gene translation [75]. In maize (*Zea mays*), dysfunction of ZmMTA causes severe arrest during embryogenesis and endosperm development [76]. MTA and MTB are indispensable for the normal ripening of the non-climacteric fruit strawberry (*Fragaria × ananassa*), regulating this process by modulating the abscisic acid (ABA) signaling pathway [77].

m^6^A writers also contribute to abiotic and biotic stress responses in plants. For example, *Arabidopsis mta* and *fip37* mutants exhibit cold-sensitive growth [78,79], and FIP37 activity is further regulated by TMK4-mediated phosphorylation [80]. In apple (*Malus domestica*), MdMTA enhances lignin deposition and the scavenging of reactive oxygen species (ROS) under drought conditions [81]. In wheat (*Triticum aestivum*), TaMTB interacts with the WYMV viral protein NIB, stabilizing viral RNA and promoting infection [82] (Fig. 1). Beyond the MTC complex, the METTL16 homolog FIONA1 installs m^6^A on U6 snRNA and a small subset of mRNAs [83], especially within coding regions, affecting their abundance and processing in *Arabidopsis*. FIONA1-mediated methylation regulates the length of the circadian period [84] and plays important roles in photomorphogenesis and the floral transition by modulating the expression of genes encoding key regulators of flowering such as CO, SOC1, FLC, SPL3, and SEP3 [85,86].

Active m^6^A demethylation is the basis for its dynamic reversible regulation. In mammals, m^6^A demethylation is catalyzed by two key enzymes: FTO (fat mass and obesity-associated protein) and ALKBH5. FTO, the first identified m^6^A demethylase, has been shown to remove internal m^6^A from mRNA [11] and certain caRNAs [13,14]. By contrast, ALKBH5 has exhibited substrate specificity toward mRNA m^6^A thus far [15]. Plants lack FTO homologs, but ALKBH-like proteins are conserved in plants. Remarkably, the heterologous expression of human *FTO* in rice and potato (*Solanum tuberosum*) led to global m^6^A demethylation and increased biomass and yield by up to 50 % [87]. In *Arabidopsis*, two m^6^A demethylases have been identified: AtALKBH9B and AtALKBH10B [23,88]. AtALKBH9B plays an important role in regulating plant responses to viral infection [88] and contributes to ABA signaling by modulating the m^6^A methylation levels of negative regulators such as *ABI1* and *BES1* [25]. AtALKBH10B regulates flowering by demethylating the transcripts of floral integrator genes, thereby influencing mRNA stability and flowering time [23]. In tomato, the demethylase SlALKBH2 targets the mRNA of *SlDML2*, a key regulator of fruit ripening, affecting its stability and, consequently, the ripening process. Notably, *SlDML2* also modulates the DNA methylation (5mC) status of the *SlALKBH2* promoter, creating a feedback regulatory loop [62]. In watermelon, ClALKBH4B demethylates the m^6^A modifications of immune-related transcripts and modulates host susceptibility to cucumber green mottle mosaic virus [89]. In response to heat stress, AtALKBH9B relocalizes to stress granules (SGs) and demethylates heat-induced transcripts such as the retrotransposon *Onsen*, facilitating their release from SGs and subsequent expression [90]. *AtALKBH10B* expression is induced by salt, osmotic stress, and stress-related plant hormones (ABA and jasmonic acid). The *alkbh10b* mutant is hypersensitive to NaCl, mannitol, drought stress, and ABA treatment, suggesting that AtALKBH9B plays a key role in abiotic-stress tolerance [[91], [92], [93]] (Fig. 1). By contrast, *ALKBH10B* mutants in cotton (*Gossypium hirsutum*) and tomato exhibit enhanced drought tolerance [94,95], suggesting that ALKBH10B-mediated m^6^A demethylation has opposite effects on drought responses across plant species.

In eukaryotes, m^6^A signals are interpreted by specific reader proteins. The most extensively characterized reader proteins are YTH domain-containing family proteins, which are divided into the YTHDF-like and YTHDC-like subgroups. These proteins recognize m^6^A-modified transcripts through a conserved aromatic pocket in the YTH domain and influence mRNA stability, splicing, and translation [[96], [97], [98]]. In *Arabidopsis*, the YTHDF-type proteins known as ECTs play major roles in development and stress responses. AtECT2, AtECT3, and AtECT4 stabilize transcripts involved in ABA signaling and contribute to leaf and trichome development [99,100]. AtECTs regulate the fate of modified transcripts via their recruitment to SGs and processing bodies. AtECT1 promotes the decay of salicylic-acid-related mRNAs [101], AtECT2 relocates to SGs under heat stress [102], and AtECT8 functions with DECAPPING5 to degrade salt-responsive transcripts and forms phase-separated condensates that mediate an m^6^A-dependent feedback loop involved in ABA signaling [103,104]. AtECT2 also binds to and destabilizes Pepino mosaic virus (PepMV) RNA to restrict infection [105].

m^6^A readers perform diverse and specialized roles in various crops. In rice, OsECT3 is required for cold tolerance and undergoes lysine acetylation that suppresses its m^6^A-binding activity [106]. In tomato, SlYTH2 inhibits fruit aroma biosynthesis by suppressing translation [107]. In apple, MhYTP2 binds to *MdMLO1*9 mRNA, enhancing resistance to powdery mildew [108]. In foxtail millet (*Setaria italica*), SiYTH1 regulates drought tolerance by modulating stomatal movement and ROS-related gene expression [109]. In addition, the *Arabidopsis* genome encodes two YTHDC-type proteins, AtECT12 and CPSF30-L. AtECT12 regulates the stability of m^6^A-modified RNA transcripts, thereby facilitating abiotic-stress responses [110]. CPSF30-L uniquely contains a YTH domain and controls alternative polyadenylation via m^6^A binding and phase separation, thereby regulating flowering and ABA responses [111,112]. In summary, YTH readers interpret m^6^A signals, leading to specific developmental, immune, and environmental responses across plant species (Fig. 1).

### 2.3 Methods for detecting m^6^A in plants

To date, most transcriptome-wide m^6^A mapping in plants has relied on antibody-based immunoprecipitation followed by sequencing (m^6^A-RIP-seq or MeRIP-seq) [113]. While widely used, this method offers only moderate resolution and relies heavily on antibody specificity. In recent years, several alternative techniques have been successfully applied to plant systems, offering improved sensitivity and specificity (Table 1).

**Table 1:** Methods for m^6^A detection in plants.

Method name	Method target	Method approach
MeRIP-seq	m^6^A-enriched regions on mRNA or other lncRNAs	Antibody-based immunoprecipitation of m^6^A-modified RNAs followed by sequencing
miCLIP	Single nucleotide m^6^A sites on mRNA	Antibody + UV crosslinking to induce reverse transcription (RT) mutations
m^6^A-REF-seq	m^6^A sites within ACA motifs	MazF/ChpBK methylation-sensitive cleavage at ACA
m^6^A-SEAL-seq	Internal m^6^A sites across the transcriptome	FTO oxidation → hm^6^A → DTT thiol-addition → chemical tagging
Oxford Nanopore Direct RNA Sequencing (DRS)	Native RNA modification signals including m^6^A	Detection of shifts in the electrical current from direct RNA threading
DENA (deep learning model for DRS)	Single-base m^6^A inferred from Nanopore signals	Deep neural network trained on wild-type vs. m^6^A-deficient ONT data
m^6^A-SAC-seq	Single-base m^6^A sites across the transcriptome	MjDim1 enzymatic labeling + chemical conversion + RT mutation signatures
CAM-seq	Single-base m^6^A sites across the transcriptome	Selective deamination of unmethylated A under mild catalytic conditions

m^6^A-SEAL-seq enables antibody-free chemical labeling of m^6^A through FTO-mediated oxidation, providing high-resolution m^6^A maps in rice without sequence-motif or delivery constraints [114]. m^6^A-REF-seq, which relies on the methylation-sensitive cleavage of ACA motifs by ChpBK or MazF endoribonucleases, has been validated in poplar (*Populus trichocarpa*), with consistent results compared to m^6^A-RIP-seq [115]. miCLIP achieves single-nucleotide resolution through UV-induced crosslinking with antibodies and has been used to validate plant m^6^A sites [116,117]. Oxford Nanopore direct RNA sequencing (DRS) offers enrichment-free detection of m^6^A from native RNA and has been successfully utilized in *Arabidopsis*, bamboo, poplar, and apple [115,116,118]. Recent developments such as DENA, a deep learning model trained on wild-type and m^6^A-deficient *Arabidopsis* lines, have further improved detection accuracy and isoform-level resolution [119]. m^6^A-SAC-seq is a recently developed chemical labeling strategy that achieves single-base resolution of m^6^A across the whole transcriptome. This technique was recently used to generate high-resolution m^6^A maps in various tissues of *Arabidopsis* and rice, identifying over 200,000 m^6^A sites across more than 20,000 genes [66]. Chemical cooperative catalysis-assisted m^6^A sequencing (CAM-seq) further expands this toolkit by enabling base-resolution identification of m^6^A through selective deamination of unmethylated adenosines under mild conditions; this technique has been successfully utilized in *Arabidopsis* [120].

Collectively, these advances provide powerful tools for exploring the plant m^6^A epi-transcriptome. As single-base resolution mapping becomes more accessible, future studies will likely uncover further regulatory roles and mechanistic connections between m^6^A and plant development, stress adaptation, and epigenetic networks. However, a key limitation of most current methods is that they are optimized for purified polyadenylated mRNAs and may not effectively capture non-polyadenylated or chromatin-associated RNA species. Addressing this limitation is critical for extending m^6^A profiling to broader RNA populations and understanding their functions.

### 2.4 Other modifications in plant mRNAs

Beyond m^6^A, other internal RNA modifications have also been detected in plants, such as N^1^-methyladenosine (m^1^A), m^5^C, N^4^-acetylcytidine (ac^4^C), pseudouridine (Ψ), 8-hydroxyguanosine (8-OHG), and 8-nitroguanosine (8-NO_2_G). However, the roles of these RNA modifications remain less well understood.

In *Petunia hybrida*, m^1^A shows tissue- and stage-specific dynamics. Transcriptome-wide profiling identified nearly 5000 m^1^A peaks across over 3000 genes in corollas, with enrichment in coding regions just after start codons, resembling patterns observed in mammals. Ethylene treatment altered the m^1^A landscape, modulating many target transcripts. Silencing of the m^1^A methyltransferase gene *PhTRMT61A* led to reduced m^1^A levels and developmental abnormalities, suggesting that m^1^A contributes to organogenesis and phytohormone signaling [121].

m^5^C-RIP-seq identified over 6000 m^5^C peaks in more than 4000 *Arabidopsis* genes; these peaks were enriched in coding regions near start and stop codons. These modifications were associated with transcripts showing low translation efficiency. The methyltransferase TRM4B mediates m^5^C deposition; its mutation disrupts mRNA stability and root development [122]. In rice, OsNSUN2 catalyzes m^5^C formation during heat stress. Loss of OsNSUN2 leads to lesion-mimic phenotypes and increased heat sensitivity. Under heat stress, OsNSUN2 promotes m^5^C methylation on mRNAs encoding photosynthesis- and detoxification-related enzymes, enhancing their translation. Without this regulation, stress responses are compromised, leading to photosystem instability and ROS accumulation [123].

Transcriptome-wide analyses in *Arabidopsis* and rice revealed ac^4^C enrichment near translation start sites and, in *Arabidopsis*, also near stop codons. ac^4^C is positively correlated with mRNA stability, splicing diversity, and translation efficiency, while it reduces the formation of RNA secondary structures [124]. During *Magnaporthe oryzae* infection in rice, ac^4^C levels increase, particularly at the third codon position, enhancing the translation of defense-related genes [125]. Recent studies have revealed the important roles of mRNA acetylation in photosynthesis and flowering. Eliminating the RNA acetyltransferase ACYR/NAT10 in *Arabidopsis* and rice reduced ac^4^C levels and suppressed the translation efficiency of key photosynthetic transcripts, thereby disrupting LIGHT-HARVESTING COMPLEX (LHC) protein homeostasis [126].

The RNA modification Ψ was recently shown to play multilayered roles in translational regulation across rRNA, tRNA, and mRNA in plants. Transcriptome-wide Ψ profiling revealed that Ψ levels are positively correlated with translation efficiency but negatively correlated with mRNA stability [127]. In rice, Ψ of chloroplast rRNA catalyzed by OsPUS1 is essential for ribosome biogenesis and cold tolerance, as the loss of OsPUS1 led to impaired chloroplast development at low temperature [128]. These findings highlight the importance of Ψ in fine-tuning translational output and stress adaptation in plants.

Oxidative and nitrative mRNA modifications contribute to post-transcriptional gene regulation during plant development and immunity. 8-OHG accumulates on specific mRNAs during normal developmental transitions, including seed germination in sunflower and wheat, where the oxidation of defined transcripts reduces the abundance of their encoded proteins [129,130]. 8-OHG formation on mRNAs also occurs rapidly in plants under stress, such as cadmium exposure in soybean (*Glycine max*) and nematode infection in *Arabidopsis* [131,132]. In addition, nitrative RNA modifications such as 8-NO_2_-G transiently accumulate on mRNAs in potato during pathogen attack and correlate with the activation of the hypersensitive response [133].

Together, these findings shed light on the mechanisms and functions of mRNA modifications, especially m^6^A, in plants. However, it is still unknown whether these modifications, particularly m^6^A, are also present and act on other RNA classes. In addition, less is known about how RNA modifications coordinate with other epigenetic mechanisms in plants.

### 2.5 Major modifications in rRNA and tRNA

rRNAs, which are among the most conserved RNA species in eukaryotes, harbor multiple chemical modifications that fine-tune ribosome stability, biogenesis, and translational output. The major ribosomal RNAs in plants, including 25S, 18S, 5.8S, and 5S rRNAs, carry diverse modifications such as m^6^A, m^5^C, m^1^A, Ψ, N7-methylguanosine (m^7^G), and several 2′-*O*-methylated nucleotides including Am, *Cm*, Gm, and Um [134]. Although m^6^A modification in plant mRNA has been well characterized, its role in rRNA remains largely unexplored. Two recent studies demonstrated that *Arabidopsis* METTL5 specifically catalyzes m^6^A deposition at position A^1771^ of 18S rRNA, a modification essential for ribosome assembly and the translation of stress-responsive genes [135,136]. Ψ is also highly abundant in plant rRNAs. Recent bisulfite-induced deletion sequencing has generated single-nucleotide-resolution Ψ maps in rice, maize, *Arabidopsis*, and soybean. These studies showed that nucleus-encoded rRNAs contain substantially more Ψ sites than chloroplast- or mitochondrion-encoded rRNAs. A considerable proportion of these Ψ sites are conserved among species, with strong correlations in Ψ stoichiometry. Ψ of rRNA plays multilayered roles in maintaining rRNA integrity and globally modulating translation efficiency in plants [127].

tRNAs, together with their RNA modifications, act as key regulators of translation by shaping codon-specific decoding, thereby influencing both the rate and efficiency of protein synthesis. Bioinformatics analyses combined with nucleoside abundance and gene expression profiling in rice and *Arabidopsis* indicated that the methylated nucleosides Am, *Cm*, m^1^A, and m^7^G are closely associated with stress responses, whereas Gm, m^5^U, and m^5^C are more strongly linked to developmental processes [137]. In rice, the Am tRNA modification increased under salt and ABA treatment; the methyltransferase OsTRM13 is responsible for catalyzing this modification, as purified OsTRM13 protein catalyzed the formation of Am on tRNA-Gly-GCC *in vitro*. OsTRM13 enhances salt tolerance in plants, as demonstrated by increased resistance in overexpression lines and reduced tolerance in RNA interference lines [138]. In *Arabidopsis*, loss of the tRNA m^5^C methyltransferase TRM4B reduced the stability of tRNA^Asp(GTC)^. *trm4b* mutants showed shorter primary roots than the wild type due to impaired cell division in the root apical meristem and displayed heightened sensitivity to oxidative stress [139].

The nucleus-localized AtTRM61/AtTRM6 complex catalyzes the methylation at N^1^ of adenosine 58 (A58) modification on tRNAᵢᴹ^e^ᵗ; the loss of either subunit abolished this activity. The resulting decrease in tRNA m^1^A led to embryo arrest and seed abortion, highlighting the essential role of this modification in maintaining tRNAᵢᴹ^e^ᵗ stability [140]. In a recent study of Ψ in the T-arm loop of tRNA in *Arabidopsis*, strong positive correlations were detected between Ψ stoichiometry and the translation efficiency of the corresponding codons, with similar but weaker associations observed in the anticodon-arm and D-arm loops. By contrast, Ψ levels in stem regions of tRNA showed no significant relationship with the translation efficiency of codons, indicating that loop-localized Ψ plays a major role in regulating protein synthesis [127].

## 3 Epigenetic regulation in plants

### 3.1 Overview of epigenetic regulation mechanisms in plants: DNA methylation, histone modifications, and chromatin remodeling

Epigenetic regulation involves DNA methylation, chromatin remodeling, RNA modifications, histone modifications, and histone variants. Among these, DNA methylation, including 5-methylcytosine (5mC) and N^6^-methyladenine (6mA), plays key roles in transcriptional regulation and genome stability in both plants and animals. By contrast, 5-hydroxymethylcytosine (5-hmC), an important oxidation derivative of 5mC in animals, has not been reliably detected in plant genomes, and its presence or function in plants remains unconfirmed. Plant genomes exhibit 5mC in the CG, CHG, and CHH contexts, with CHH methylation being re-established *de novo* by the plant-specific RdDM pathway [30,31,34]. The maintenance of DNA methylation in different sequence contexts is mediated by distinct methyltransferases. CG methylation is maintained by MET1 [[141], [142], [143]], CHG methylation depends on CMT2 and CMT3 [144], and CHH methylation mainly relies on DRM2 or CMT2 in *Arabidopsis* and rice [34,145]. Active DNA demethylation in *Arabidopsis* is carried out by DNA glycosylases such as DME, ROS1, DML2, and DML3, which remove 5mC via base excision repair [146]. In rice, DNG701/704 and DNG702/703 are homologous to DML2 and ROS1, respectively, but no DME homolog has been identified [[147], [148], [149], [150]]. In addition to 5mC, 6mA has emerged as a stress-responsive epigenetic mark in plants. Plant ALKBH1 orthologs possess 6mA demethylase activity, particularly on single-stranded DNA [[151], [152], [153], [154]], as similar activities have been observed in *E. coli* AlkB and human ALKBH1 [155,156].

Histone proteins, forming the cores of nucleosomes, undergo diverse post-translational modifications, including methylation, acetylation, phosphorylation, and ubiquitination, which regulate chromatin structure and gene activity. Repressive histone marks such as H3K9me3 and H3K27me3 are enriched at heterochromatin and silent gene loci, while active marks such as H3K4me3, H3K27ac, and H3K36me3 are present at transcriptionally active loci [157]. Histone lysine methylation is primarily catalyzed by SET-domain group (SDG) proteins in plants. SDG proteins, which are classified into the SUVH/SUVR, E(z)-like, TRX-like, and ASH1-like families, target distinct histone residues. Polycomb Repressive Complex 2 (PRC2), containing E(z) homologs, deposits H3K27me3 and regulates developmental transitions, vernalization, and responses to stress and phytohormones [52,153,158,159]. Histone demethylation is mediated by LSD- and JMJ-family proteins. In *Arabidopsis*, JMJ11/12/13/30/32 remove H3K27me3 and regulate flowering time in response to environmental cues [41,159]. In rice, JMJ705 demethylates H3K27me3, contributing to disease resistance, meristem maintenance, and energy regulation [38,39].

Chromatin remodelers regulate chromatin accessibility through the ATP-dependent repositioning, eviction, or restructuring of nucleosomes and can also recruit other epigenetic regulators to influence DNA methylation and the histone modification landscape [160,161]. In plants, major families of chromatin remodeling factors include SWItching defective/Sucrose Non-Fermenting (SWI/SNF), Imitation SWItch (ISWI), Chromodomain Helicase DNA-binding (CHD), INOsitol requiring 80 (INO80), and Microrchidia (MORC). Studies in *Arabidopsis*, rice, and maize have demonstrated that mutations in these remodelers not only alter chromatin accessibility but also create distinct chromatin environments that affect the deposition of DNA methylation and histone modifications [33,47,[162], [163], [164], [165]].

### 3.2 Crosstalk between mRNA m^6^A modification and other epigenetic factors in plants

Although studies on the interactions between m^6^A and chromatin modifications in plants remain limited, recent findings have begun to reveal key regulatory connections. In both plants and animals, H3K36me can recruit the m^6^A methyltransferase complex. In *Arabidopsis*, FIP37 is recruited to H3K36me-marked regions, facilitating co-transcriptional m^6^A deposition on nearby transcripts and linking histone with RNA modifications [166]. Notably, while animals mainly use H3K36me3 for this purpose, plants rely more on H3K36me2 [167]. A recent study showed that the plant-specific protein NERD (NEEDED FOR RDR2-INDEPENDENT DNA METHYLATION) is a crucial component of the *Arabidopsis* m^6^A methyltransferase complex. NERD stabilizes the core writers MTA and MTB, promoting the addition of m^6^A on nascent RNAs and globally repressing gene expression. At the *FLC* locus, NERD interacts with the histone methyltransferase SDG8 to modulate H3K36me3 levels, thereby coordinating the transcriptional repression of *FLC* via both RNA and histone modification pathways [168]. Additionally, recent work in maize identified direct crosstalk between m^6^A and DNA methylation. The m^6^A methyltransferase ZmMTA interacts with ZmDDM1, a chromatin remodeler essential for 5-methylcytosine (5mC) maintenance. Genes marked by m^6^A tend to show higher levels of DNA methylation. Disrupting ZmMTA resulted in severe developmental arrest and reduced CHH methylation around m^6^A-modified gene promoters, suggesting that m^6^A might influence local DNA methylation patterns. By contrast, the loss of ZmDDM1 did not affect ZmMTA function, indicating a unidirectional regulatory effect [76].

Together, these findings reveal emerging connections between m^6^A and chromatin-level regulation in plants. However, the interplay between m^6^A and other epigenetic modifications remains largely unexplored. Further study is needed to fully understand how m^6^A cooperates with histone and DNA modifications to regulate gene expression in plants. By contrast, studies in mammals have uncovered a vast repertoire of caRNAs, including both coding and non-coding transcripts. These RNAs are either retained at their sites of transcription or recruited to distant genomic loci. Together with associated RNA-binding proteins, caRNAs contribute to transcriptional regulation, chromatin organization, enhancer–promoter interactions, and the formation of nuclear bodies [169]. Among these caRNAs, lncRNAs exhibit distinct spatial localization and functional specificity in modulating chromatin states [170]. Further investigation into the chemical modifications of caRNAs, such as methylation, will provide deeper insights into chromatin-based regulatory mechanisms.

## 4 Definition, classification, and genomic functions of caRNAs

RNAs capable of interacting with chromatin include nascent RNAs, lncRNAs, circular RNAs (circRNAs), small nuclear RNAs (snRNAs), small nucleolar RNAs (snoRNAs), enhancer RNAs (eRNAs), promoter-associated RNAs (paRNAs), antisense RNAs (asRNAs), and repeat-derived RNAs. Nascent transcripts, such as pre-mRNAs and pre-ncRNAs, can form R-loops or function as scaffolds at transcription sites, shaping chromosomal architecture and opposing chromatin condensation [171,172]. lncRNAs and circRNAs (>200 nt) frequently associate with chromatin modifiers and epigenetic regulators [173]. snRNAs and snoRNAs participate in splicing and RNA modification but also form ribonucleoprotein (RNP) complexes that can interact with chromatin [174]. Regulatory RNAs such as eRNAs, paRNAs, and asRNAs are typically transcribed from specific genomic elements and exert local effects on chromatin [175,176]. Advanced high-throughput technologies, such as MARGI [177], ChAR-seq [178], RADICL-seq [179], and GRID-seq [180], have been used to map RNA–chromatin interactions at genome-wide resolution, revealing diverse and novel caRNAs beyond classical examples like Xist. These studies highlight the pervasive roles of caRNAs in transcriptional regulation, nuclear architecture, and pathogenesis.

### 4.1 Chromatin-associated RNAs in plants

Increasing evidence indicates that caRNAs, such as small RNAs, lncRNAs, and the RNA strands within R-loops, participate in classical, essential epigenetic pathways in plants. These caRNAs help regulate gene expression by modulating genome architecture and chromatin state.

**Fig. 2:**
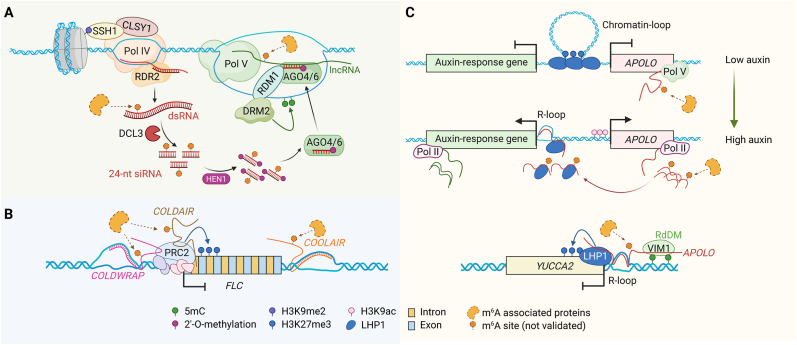
CaRNAs and epigenetic modifications in plants. **A** In the RdDM pathway, caRNAs transcribed by Pol V serve as scaffolds for AGO–siRNA complexes, guiding DRM2-mediated *de novo* DNA methylation and transcriptional silencing. **B** Multiple lncRNAs regulate the chromatin state at the *FLC* locus: *COLDWRAP* and *COLDAIR* facilitate PRC2 recruitment and H3K27me3 deposition, while *COOLAIR* modulates transcription through antisense-mediated and altered RNA structure. **C** The lncRNA *APOLO* regulates chromatin conformation and coordinates multiple epigenetic pathways during auxin signaling. *APOLO* modulates chromatin loop dynamics and forms R-loops, thereby influencing the deposition or removal of key chromatin marks, including H3K27me3, H3K9ac, and DNA methylation. At the *YUCCA2* locus, APOLO-associated R-loops enable VIM1-dependent RdDM recruitment, reinforcing transcriptional repression. While only a few examples are known, accumulating evidence suggests that caRNAs widely interface with chromatin modifiers to shape transcriptional programs that regulate plant development and stress adaptation. Figure was created with BioRender.com.

Small RNAs are central to the RdDM pathway, which establishes *de novo* cytosine methylation in plants. 24-nt siRNAs are generated from repetitive or transposon-rich regions via RNA polymerase IV (Pol IV), RNA-dependent RNA polymerase 2 (RDR2), and Dicer-like 3 (DCL3). These siRNAs are loaded into ARGONAUTE proteins (AGO4/6), which guide the complex to scaffold RNAs transcribed by RNA polymerase V (Pol V), allowing DRM2 to catalyze CHH methylation [33] (Fig. 2A). In rice, OsDRM2 is essential for generating 24-nt siRNAs and CHH methylation [34], while mutating *OsDDM1* increased the production of heterochromatic siRNAs and lncRNAs. These findings indicate that both factors reinforce the RdDM pathway through coordinated effects on small RNA biogenesis and chromatin structure [48]. Recent studies have also identified a class of DCL3-dependent 24-nt microRNAs that are loaded into AGO4-clade proteins and can direct DNA methylation at both their own loci and *trans*-targets [181].

Plant genomes encode numerous lncRNAs that associate with chromatin to regulate the expression of genes involved in development and stress adaptation. These transcripts act in *cis* or *trans* by recruiting chromatin modifiers or forming R-loops. Vernalization-induced lncRNAs such as *COLDAIR*, *COLDWRAP*, and *COOLAIR* recruit PRC2 to deposit H3K27me3 at the *FLC* locus, enabling stable gene silencing and flowering [[50], [51], [52]] (Fig. 2B). The lncRNA *APOLO* modulates auxin-responsive gene networks through the formation of R-loops and the recruitment of LHP1 [182]. *APOLO* also interacts with VIM1 to connect histone modification with DNA methylation dynamics during thermomorphogenic responses [53] (Fig. 2C). Drought-responsive lncRNAs further expand this regulatory landscape. *DANA2* positively regulates the expression of the H3K9 demethylase JMJ29 by interacting with the transcription factor ERF84 [183]. Under drought stress, *DANA1* interacts with DIP1**,** promoting histone deacetylation at *CYP707A1/2* loci via the recruitment of HDA9 and PWWP3 (a PEAT complex component), thereby repressing ABA catabolism and enhancing drought resistance [184].

R-loops, a widespread genomic feature in plants, are co-transcriptionally generated three-stranded structures composed of an RNA:DNA hybrid and a displaced single DNA strand [185]. R-loops are generally associated with low levels of DNA methylation, particularly in the CG context, and often colocalize with specific histone modifications, suggesting that they might help shape local chromatin environments. Functional studies have highlighted how R-loop resolution is integral to epigenetic regulation: DDM1 resolves R-loops in heterochromatic regions, facilitating H2A.W incorporation and the exclusion of H2A.Z, thereby promoting proper heterochromatin formation [49]. *Arabidopsis* Topoisomerase 1 prevents excessive R-loop overaccumulation that would otherwise disrupt auxin distribution and impair root development [186]. R-loops at the *FLC* locus also help regulate flowering time and participate in stress responses by transiently modulating transcriptional dynamics [185].

Together, these findings underscore how chromatin-associated RNAs in plants exert essential, finely tuned control over development and stress adaptation through diverse mechanisms. However, whether these caRNAs themselves carry chemical modifications, and how such modifications might influence their chromatin functions, remain largely unexplored, representing an important frontier for future research.

### 4.2 caRNA modifications in mammals

While most studies on RNA modifications in plants have focused on mRNAs, understanding broader gene regulatory networks requires attention to RNA–chromatin interactions. In contrast to the limited knowledge in plants, animal studies have established caRNAs as crucial regulators of genome structure and gene expression. These RNAs are not only physically tethered to chromatin but also undergo dynamic chemical modifications that influence their localization, stability, and interactions with the chromatin-modifying machinery. These insights from animal models provide a conceptual foundation for exploring the roles of modified caRNAs in regulating chromatin in plants.

### 4.3 Modifications on caRNAs and their functional impact

Chromatin-associated RNAs are key substrates of epitranscriptomic modifications, particularly m^6^A. METTL3, the catalytic subunit of the m^6^A writer complex, installs m^6^A on nascent RNAs at promoters independently of METTL14, promoting their translation and contributing to oncogenic programs [187]. In embryonic stem cells, METTL3 deposits m^6^A on caRNAs, including promoter-associated RNAs, eRNAs, and repeat RNAs. These modifications are recognized by YTHDC1, which directs their degradation via the nuclear exosome-targeting complex. Loss of METTL3 or YTHDC1 resulted in the accumulation of caRNA, increased chromatin openness, and transcriptional upregulation [55]. YTHDC2, another nuclear reader, binds to m^6^A-modified *HERV-H* RNAs and regulates stem cell fate [58]. Conversely, the demethylase FTO removes m^6^A from LINE1 caRNAs. The deletion of FTO increased m^6^A levels, promoted YTHDC1 binding, and accelerated the decay of *LINE1* RNA [13,14]. Another demethylase, ALKBH5, regulates the chromatin-associated lncRNA *CARMN* in colorectal cancer by reversing m^6^A methylation and preventing YTHDF2/3-mediated degradation, thereby suppressing tumor progression [188].

Beyond m^6^A, m^5^C modifications on caRNAs have been characterized using m^5^C-TAC-seq, revealing enrichment in introns, short interspersed nuclear elements (SINEs), and transcripts with low levels of translation. NSUN2 and NSUN5 catalyze m^5^C deposition [189]. Moreover, TET2 oxidizes RNA m^5^C, altering histone marks and chromatin structure, especially in leukemia stem cells [59].

### 4.4 Emerging understanding of RNA–chromatin crosstalk: mechanistic interplay and biological implications

caRNAs serve as scaffolds, guides, or decoys that interface with chromatin-modifying enzymes and transcriptional regulators. Epitranscriptomic marks on these RNAs further modulate their stability, localization, and capacity to influence the chromatin state [54].

**Fig. 3:**
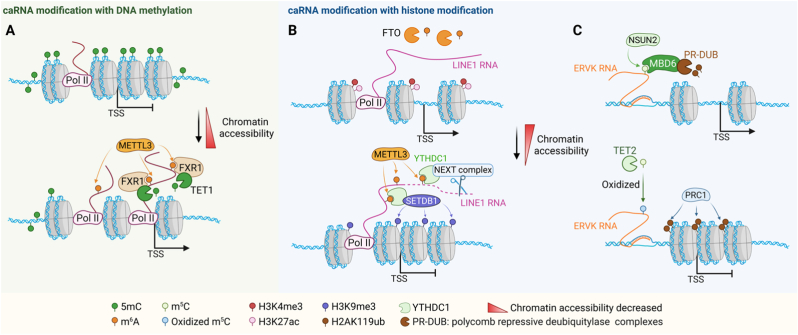
Crosstalk between caRNA modifications and the epigenetic regulation of chromatin. **A** METTL3 installs m^6^A marks on caRNAs. The m^6^A reader protein FXR1 binds to these modified RNAs and recruits the DNA demethylase TET1 to gene body regions, facilitating the removal of CG methylation. This results in increased chromatin accessibility and the activation of transcription. **B** Chromatin-associated *LINE1* RNAs undergo m^6^A methylation, which can be reversed by the demethylase FTO. m^6^A-marked *LINE1* RNAs are recognized by the reader YTHDC1, which promotes the recruitment of the NEXT complex and SETDB1, contributing to the deposition of the repressive H3K9me3 mark and chromatin condensation. **C** m^5^C modifications on *ERVK* RNAs are deposited by NSUN2 and can be oxidized by TET2. Modified *ERVK* RNA interacts with MBD6 and the PR-DUB complex, influencing the removal of H2AK119ub and regulating PRC1 occupancy. These modifications collectively reshape chromatin accessibility and transcriptional potential. Figure was created with BioRender.com.

It is becoming clear that caRNAs not only undergo m^6^A modification but also actively participate in shaping local DNA methylation landscapes through direct RNA-DNA crosstalk. The METTL3-METTL14 complex can recruit DNMT1, establishing 5mC on gene bodies and enhancing transcription, while m^6^A simultaneously promotes RNA turnover to fine-tune the expression of genes involved in differentiation [190]. YTHDC2 recognizes m^6^A-marked HERV-H RNAs and recruits TET1 to remove 5mC and activate chromatin at transposable elements, modulating pluripotency [58]. METTL3 installs m^6^A on nascent chromatin-associated RNAs, which are recognized by the reader protein FXR1. FXR1 recruits the DNA demethylase TET1 to gene bodies, where TET1 catalyzes the removal of CG methylation, converting highly methylated regions into a hypomethylated state. This demethylation increases chromatin accessibility and facilitates transcriptional activation [57] (Fig. 3A).

caRNAs also regulate chromatin via histone modification pathways. caRNA enrichment is positively correlated with H3K27ac/H3K4me3 (active marks) and negatively correlated with H3K9me3 (repressive marks) [177]. METTL3 installs m^6^A on caRNAs, including promoter- and enhancer-derived transcripts, which are recognized by the reader YTHDC1 and targeted for degradation. The loss of m^6^A led to caRNA accumulation, increased H3K4me3 and H3K27ac, enhanced chromatin accessibility, and widespread transcriptional activation [54,55] (Fig. 3B). METTL3 also maintains the integrity of heterochromatin at IAP retroelements through RNA methylation and interactions with SETDB1/TRIM28, promoting H3K9me3 deposition [56]. By contrast, FTO-mediated m^6^A demethylation stabilizes *LINE1* caRNAs and maintains open chromatin with high levels of H3K4me3 and H3K27ac. Deleting *FTO* increased m^6^A levels on *LINE1* transcripts, promoting YTHDC1-dependent degradation and SETDB1 recruitment, which in turn induced H3K9me3 accumulation and chromatin compaction [13,191] (Fig. 3B). In parallel, m^5^C modification is also associated with the regulation of chromatin. TET2 oxidizes m^5^C on retrotransposon-derived caRNAs, thereby preventing recognition by the reader MBD6. This blocks MBD6-driven H2AK119ub deubiquitination and preserves transcriptional repression in hematopoietic stem cells [59] (Fig. 3C). Collectively, these findings underscore the roles of caRNAs as epigenetic integrators that mediate the communication among RNA, DNA, and histone by regulating methylation dynamics, providing multilayered control of gene expression and chromatin states.

## 5 Perspectives

RNA modifications have emerged as important regulators of chromatin state and gene expression, with caRNAs such as lncRNAs, R-loops, and small RNAs playing essential roles in transcriptional regulation and epigenetic reprogramming. In animal systems, modifications such as m^6^A and m^5^C on caRNAs have been shown to influence stem cell fate, suppress tumor progression, and regulate chromatin accessibility in leukemia stem cells. Notably, a recent study in Arabidopsis revealed that chromatin-associated retrotransposon RNAs carry m6A marks deposited by FIP37 and VIR, which are recognized by CPSF30-L and ECT12 to promote heterochromatin formation and transcriptional silencing; CPSF30-L further recruits SUVH4/5/6 and ATXR5/6 to establish H3K9me2 and H3K27me1 at these m6A-marked loci [192]. However, similar mechanistic insights across plant species remain scarce.

Despite the growing recognition of the roles of caRNAs in gene regulation, studies in plants remain limited. This is partly due to the lack of plant-adapted technologies for profiling RNA–chromatin interactions on a genome-wide scale. Moreover, plant genomes are highly repetitive, with abundant transposable elements and strong tissue-specific expression, complicating the identification and interpretation of caRNAs. The low abundance and dynamic nature of many non-coding caRNAs also pose technical barriers to functional characterization. As a result, most studies have focused on well-known examples such as lncRNA-mediated Polycomb recruitment or siRNA-guided DNA methylation, while the landscape of caRNA modifications in plants remains largely unexplored.

Future research should prioritize the development of tools to map and modify caRNAs in plant genomes at cell-type resolution. Single-base resolution mapping of caRNA-associated modifications, such as m^6^A, will be crucial for uncovering how these marks affect caRNA stability, chromatin binding, and transcriptional control. From an applied perspective, elucidating the caRNA regulatory layer in plants could improve our understanding of key developmental and adaptive traits. For instance, dissecting how caRNAs modulate chromatin during vernalization could support the genetic improvement of flowering time and yield in crops such as wheat. Additionally, targeting caRNA modifications could lead to strategies that enhance genome stability and stress resilience, advancing molecular breeding for climate-adaptive agriculture.

However, a major limitation in understanding m^6^A of caRNAs in plants is the lack of suitable methods for analysis. Most current methods have limited sensitivity or resolution in plant systems. Recent advances such as GLORI 2.0 [193], which enables antibody-free, single-base resolution mapping of RNA m^6^A, hold promise for overcoming these challenges. Integrating such technologies with plant chromatin fractionation and RNA profiling will be essential for unveiling the functional landscape of caRNA modifications.

## CRediT authorship contribution statement

**Xinran Zhang:** Writing – review & editing, Writing – original draft. **Jiangbo Wei:** Writing – review & editing, Writing – original draft.

## Declaration of competing interest

The authors declare that they have no known competing financial interests or personal relationships that could have appeared to influence the work reported in this paper.

## Data Availability

No data was used for the research described in the article.
